# Comparison of 454 Ultra-Deep Sequencing and Allele-Specific Real-Time PCR with Regard to the Detection of Emerging Drug-Resistant Minor HIV-1 Variants after Antiretroviral Prophylaxis for Vertical Transmission

**DOI:** 10.1371/journal.pone.0140809

**Published:** 2015-10-15

**Authors:** Andrea Hauser, Claudia Kuecherer, Andrea Kunz, Piotr Wojtek Dabrowski, Aleksandar Radonić, Andreas Nitsche, Stefanie Theuring, Norbert Bannert, Julius Sewangi, Paulina Mbezi, Festo Dugange, Gundel Harms, Karolin Meixenberger

**Affiliations:** 1 HIV and other Retroviruses, Robert Koch-Institute, Berlin, Germany; 2 Institute of Tropical Medicine and International Health, Charité - Universitätsmedizin Berlin, Berlin, Germany; 3 Regional AIDS Control Program Mbeya Region, Ministry of Health and Social Welfare, Dar es Salaam, Tanzania; 4 PMTCT Service Mbeya Region, Ministry of Health and Social Welfare, Dar es Salaam, Tanzania; 5 Kyela District Hospital, Ministry of Health and Social Welfare, Dar es Salaam, Tanzania; 6 Centre for Biological Threats and Special Pathogens 1, Highly Pathogenic Viruses, Robert Koch-Institute, Berlin, Germany; Centro de Biología Molecular Severo Ochoa (CSIC-UAM), SPAIN

## Abstract

**Background:**

Pregnant HIV-infected women were screened for the development of HIV-1 drug resistance after implementation of a triple-antiretroviral transmission prophylaxis as recommended by the WHO in 2006. The study offered the opportunity to compare amplicon-based 454 ultra-deep sequencing (UDS) and allele-specific real-time PCR (ASPCR) for the detection of drug-resistant minor variants in the HIV-1 reverse transcriptase (RT).

**Methods:**

Plasma samples from 34 Tanzanian women were previously analysed by ASPCR for key resistance mutations in the viral RT selected by AZT, 3TC, and NVP (K70R, K103N, Y181C, M184V, T215Y/F). In this study, the RT region of the same samples was investigated by amplicon-based UDS for resistance mutations using the 454 GS FLX System.

**Results:**

Drug-resistant HIV-variants were identified in 69% (20/29) of women by UDS and in 45% (13/29) by ASPCR. The absolute number of resistance mutations identified by UDS was twice that identified by ASPCR (45 vs 24). By UDS 14 of 24 ASPCR-detected resistance mutations were identified at the same position. The overall concordance between UDS and ASPCR was 61.0% (25/41). The proportions of variants quantified by UDS were approximately 2–3 times lower than by ASPCR. Amplicon generation from samples with viral loads below 20,000 copies/ml failed more frequently by UDS compared to ASPCR (limit of detection = 650 copies/ml), resulting in missing or insufficient sequence coverage.

**Conclusions:**

Both methods can provide useful information about drug-resistant minor HIV-1 variants. ASPCR has a higher sensitivity than UDS, but is restricted to single resistance mutations.

In contrast, UDS is limited by its requirement for high viral loads to achieve sufficient sequence coverage, but the sequence information reveals the complete resistance patterns within the genomic region analysed. Improvements to the UDS limit of detection are in progress, and UDS could then facilitate monitoring of drug-resistant minor variants in the HIV-1 quasispecies.

## Introduction

Antiretroviral regimens for the prevention of mother-to-child transmission (PMTCT) of HIV have a proven efficacy in resource-limited countries. However, a major drawback of such temporary regimens is the emergence of resistant HIV-1 strains. This was extensively shown for the nevirapine single-dose (NVP-SD) regimen [1]. The implementation of the 2006 WHO-recommended triple antiretroviral regimen consisting of antenatal mono-administration of zivoduvine (AZT), NVP-SD at labor onset, and AZT plus lamivudine (3TC) for one week postpartum was assumed to reduce the development of drug resistance [1–3].

Drug resistant variants in HIV-1 protease and reverse transcriptase (RT) are routinely detected by Sanger population sequencing [4]. However, only mutant variants present at levels above 20% in the viral quasispecies of the patient can be detected by this method [5, 6], and more sensitive methods have revealed the frequent presence of drug-resistant variants in the virus population at frequencies lower than 20%. The sensitive methods were based on real-time PCR using mutant-specific oligonucleotides (allele-specific real-time PCR, ASPCR) with detection limits below 1% [7–9].

Using a highly sensitive ASPCR, we recently reported the emergence of drug-resistant minor HIV-1 variants in the plasma of 40% (20/50) of Tanzanian women following the 2006 WHO recommended PMTCT regimen [10]. In 70% of these women, resistant virus variants were present as a minority at frequencies below 5% of the total virus population. ASPCR was performed for the most common and frequent key resistance mutations: K70R selected early and transiently by AZT and T215Y/F selected by AZT, K103N and Y181C selected by NVP, and the M184V mutation selected by 3TC. The disadvantage of ASPCR is that a specific PCR-assay has to be established for each resistance position of interest, which limits the number of mutations that can be analysed and does not allow the detection of additional resistance-associated mutations present in the same genome. A more recently developed "ultra-deep sequencing" (UDS) method is also able to detect and quantify viral minorities [11, 12]. In contrast to ASPCR, UDS provides information about all mutations that differ from wild type in the genomic region analysed. This method has been used for drug-resistance testing in the HIV-1 protease [13–17], RT [13–20], and integrase [21] or to predict the HIV-1 co-receptor usage [22, 23] with sensitivities of less than 1%.

The aim of the present study was to directly compare the performance of UDS and ASPCR using a back-up plasma sample of the same sampling date from those pregnant women.

## Materials and Methods

### Ethics Statement

Ethical approval was obtained from the local Mbeya Medical Research and Ethics Committee, the National Institute for Medical Research of Tanzania and the ethical committee of Charité –Universitätsmedizin Berlin in Germany. Informed written consent was obtained from all participants involved in the study.

### The study cohort

In a previous study, 20/50 women who had taken antenatal AZT, NVP-SD at labor onset and AZT/3TC for 7 days postpartum were found to carry at least one HIV-1 drug resistance mutation in samples taken at delivery and until 12–16 week postpartum by applying ASPCR. The study revealed HIV-variants with the AZT-selected mutations in nine (18%) women, NVP-selected mutations in seven (14%) women, 3TC-selected mutation in one (2%) woman, and dual- or multi-resistant HIV-populations selected by NVP and 3TC, by AZT and 3TC, or by AZT, NVP, and 3TC in one woman (2%), respectively. In 7/20 (35%) women, HIV-1 resistance mutations were also detected by Sanger sequencing [10]. These 20 women with ASPCR-detected mutations, and 14 additional women in whom ASPCR key resistance mutations were not identified (34 women in total) were included in this sub-study to compare the previous ASPCR results with those gained by UDS. HIV RNA was extracted for UDS from 45 postpartum back-up samples (1–4 samples per woman) (Table 1). Viral loads were determined by TaqMan real-time PCR of the HIV-1 LTR genomic region according to the method published by Cleland *et al*. [24]. Back-up samples with a viral load below 1,000 copies/ml (n = 4) were excluded to minimize stochastic effects of sampling variation [14] and failure of amplicon generation [25, 26]. Finally, 41 maternal back up samples of 31 mothers (5x delivery, 2x 1–2 weeks, 29x 4–6 week, 5x 12–16 weeks) were analysed by UDS. HIV-1 subtype C was identified in 68% (21/31) and subtype A1 in 32% (10/31) of the women (Table 1).

**Table 1:** Samples included into the sub-study.

Pat No.*	Sub type	Follow up	Sanger Sequence	ASPCR Resistance Mutation; %						Sample No	Viral load (c/ml)
1	C	del	K70K/R	K70R	13	-	-	-	-	1	1.13E+03
2	C	del	K70K/R	K70R	11	-	-	-	-	2	3.26E+03
		4w	wt	wt	-	-	-	-	-	3	5.24E+04
3	A1	del	K70K/R	K70R	14	-	-	-	-	4	1.42E+04
		4w	wt	K70R	5.4	-	-	-	-	5	2.21E+04
4	A1	del	K70K/R	K70R	28	-	-	-	-	6	2.40E+04
		4w	K70K/R	K70R	14	-	-	-	-	7	4.63E+04
5	C	del	wt	K70R	2.0	T215F	0.5	-	-	8	3.47E+04
		1w	K65K/R	T215F	0.5	-	-	-	-	9	1.42E+03
		4w	wt	K70R	2.3	-	-	-	-	10	5.44E+04
		12w	wt	T215F	0.7	-	-	-	-	11	8.46E+04
6	A1	4w	wt	wt	-	-	-	-	-	12	1.87E+04
7	A1	1w	wt	K103N	10	-	-	-	-	/	<1.0E+03
		4w	wt	Y181C	0.8	-	-	-	-	/	<1.0E+03
8	C	4w	wt	K103N	1.3	-	-	-	-	13	1.39E+05
9	C	1w	wt	M184V	0.6	-	-	-	-	14	1.47E+03
		4w	wt	K103N	3.4	-	-	-	-	15	1.65E+04
10	A1	4w	wt	K70R	4.9	-	-	-	-	16	1.23E+04
11	C	4w	wt	K70R	2.7	-	-	-	-	17	3.94E+03
12	C	4w	wt	T215F	0.8	-	-	-	-	18	1.07E+03
13	C	4w	wt	T215Y	3.9	-	-	-	-	19	3.50E+04
14	A1	4w	wt	K103N	2.1	-	-	-	-	20	1.23E+03
15	C	4w	wt	K103N	3.4	-	-	-	-	21	4.69E+03
16	C	4w	K103K/N; Y181Y/C; V106V/A	M184V	0.6	K103N	36	Y181C	20	22	4.47E+03
		13w	K103K/N	K103N	12	K70R	4.0	-	-	23	7.00E+03
17	D	12w	wt	K103N	0.2	-	-	-	-	/	<1.0E+03
18	C	12w	wt	Y181C	0.4	-	-	-	-	24	6.60E+03
19	C	4w	wt	wt	-	-	-	-	-	25	9.60E+04
		12w	G190G/A	Y181C	1.5	-	-	-	-	26	6.50E+03
20	C	4w	wt	wt	-	-	-	-	-	27	4.67E+05
		12w	wt	M184V	0.6	-	-	-	-	28	2.76E+04
21	A1	4w	wt	wt	-	-	-	-	-	29	7.92E+03
22	A1	4w	wt	wt	-	-	-	-	-	30	4.87E+04
23	C	4w	wts	wt	-	-	-	-	-	31	5.74E+04
24	A1	4w	wt	wt	-	-	-	-	-	32	1.63E+05
25	A1	4w	wt	wt	-	-	-	-	-	33	4.68E+05
26	C	4w	wt	wt	-	-	-	-	-	34	7.00E+03
27	C	4w	wt	wt	-	-	-	-	-	35	2.48E+03
28	C	6w	wt	wt	-	-	-	-	-	36	1.17E+05
29	A1	4w	wt	wt	-	-	-	-	-	37	5.32E+04
30	C	4w	wt	wt	-	-	-	-	-	38	1.75E+04
31	C	4w	wt	wt	-	-	-	-	-	39	4.63E+03
32	C	4w	wt	wt	-	-	-	-	-	40	9.13E+03
33	C	4w	wt	wt	-	-	-	-	-	41	7.86E+04
34	A1	4w	wt	wt	-	-	-	-	-	/	<1.0E+03

* according to Hauser *et al*. [10]

del delivery; w week; c/ml copies per ml; wt wild type sequence (Sanger); wt no ASPCR key resistance mutation detected

### Allele-specific real-time PCR

HIV-1 subtype A-, D-, and C-specific ASPCR was established for seven resistance-associated key mutations selected by AZT (K70R, T215Y/F), 3TC (M184V), and NVP (K103N, Y181C) with detection limits below 1% as described by Hauser *et al*. [10, 27].

### Amplicon-based UDS

We participated in two international multicentre collaborative studies designed to validate two versions of an amplicon-based 454 ultra-deep pyrosequencing assay for HIV-1 drug resistance tested on the GS FLX System [11, 12]. In both studies we also analysed additional samples in a so-called 'researcher run'. Finally, forty-one postpartum plasma back-up samples from the study cohort described above were analysed in a researcher run of the follow-up international multicentre collaborative study [11]. Subtype-generic barcoded primers to amplify protease and RT and most of the reagents were obtained from 454 Life Sciences/Roche as part of the study. GS FLX Titanum Chemistry (Roche, Mannheim, Germany) was used, achieving read lengths of up to 400–500 bases. A 500 μl aliquot of each plasma back-up sample was available, and viral RNA was extracted according to manufacturer's instructions (Viral RNA-Extraction Kit, Qiagen, Hilden, Germany). Following the study protocol, 13 μl of RNA (130 μl plasma equivalent) were reverse transcribed (Transcriptor Reverse Transcriptase/Protector RNase Inhibitor/Ribonuclease H, Roche, Mannheim, Germany) using the Roche 4R- and 5R-primers, resulting in two overlapping cDNA fragments of the HIV protease and RT coding region (nt 2200–3400 of HXB2, Acc. No. K03455). Three microliter of each of the two cDNAs were used in subsequent PCRs (FastStart High Fidelity PCR System, Roche, Mannheim, Germany) with primers provided in ready-to-use microtiter plates. Six overlapping amplicons, named RTP1 to RTP6, were generated by PCR spanning protease codons 10–99 and RT codons 1–251. Amplicon sizes including adapters and MID were as follows: RTP1 = 419 bp, RTP2 = 510 bp, RTP3 = 400 bp, RTP4 = 599 bp, RTP5 = 558 bp and RTP6 = 434 bp [11]. After amplification, PCR products at concentrations below 5 ng/μl (Quant-iT^TM^ PicoGreen^®^dsDNA Reagent and Kit, Invitrogen, Darmstadt, Germany) were further analysed (Agilent 2100 Bioanalyzer, Agilent Technologies, Böblingen, Germany). PCR products with a molar ratio of primer-dimer to amplicon above 1:3 were excluded from UDS. Subsequently, amplicons RTP1-6 from each sample were pooled in equimolar proportions. Missing RTPs were compensated with one or two overlapping amplicons since all regions were covered by more than one amplicon. Clonal amplification on beads (emulsion PCR), bead isolation (breaking), and sequencing were performed according to manufacturer’s protocol for the GS FLX System (454 Life Sciences/Roche). Resulting reads were matched to the protease-RT-sequence of HXB2 (Acc. No. K03455) using the software GS Amplicon Variant Analyzer (version 2.9, 454 Life Sciences/Roche). To account for the combined error rate of amplicon-based UDS [28–30], a cut-off of 1% [11, 12] represented by at least 10 mutant reads balanced between the forward and reverse directions [16, 25] was applied to define valid unknown drug resistant minor HIV-1 variants. To compare directly UDS and ASPCR for six known key resistance mutations analysed by ASPCR (K70R, K103N, Y181C, M184V, T215Y/F), we also permitted a cut-off of less than 1% if at least 10 mutant reads balanced between the forward and reverse directions were present.

The suitability of these settings was confirmed by analysing the sensitivity of detection of seven resistance mutations in the RT as well as the error rate for all drug resistance mutations according to the IAS 2014 list [31]. This was done using recombinant HIV-1 from the wild-type pNL4.3 clone (Acc. No. M19921) and a mutant pNL4.3 derived clone that harbours seven resistance mutations in the RT (M41L, A62V, A98G, K103N, V118I, L210W, T215Y). Cloning and generation of the recombinant virus has been described previously [10, 32]. By analysing five samples of recombinant wild-type virus NL4.3 with viral loads ranging from 10^3^ to 10^9^ copies/ml, we found the highest error rate for unknown drug resistance mutations detected in our sample panel to be 0.25% for E138K. The error rate of the six key resistance mutations analysed by ASPCR was 0% each. Each of the seven RTI mutations were detected at levels above their natural specific error rates in mixtures of the wild-type recombinant HIV-1 containing 0.5% to 50% of the mutant (viral load 10^9^ copies/ml). The lowest proportion quantified was 0.32% T215Y in the 0.5% mixture.

For statistical analysis two sided Fisher’s exact test was applied and p-values <0.05 were considered to be significant.

## Results

### Amplicon-based UDS

A median viral load of 17,533 copies/ml (IQR 4,687–53,158) was determined for the 41 maternal back up samples of 31 women. PCR resulted in two to six amplicons (RTPs) per sample for 38/41 samples, while amplification failed totally for three samples (samples No. 14, 24, 26; Table 2) with low viral loads (1,470/6,600/6,500 copies/ml, respectively). Thus, 38 samples of 30 women were further analysed (Table 2).

**Table 2:** Outcome of UDS amplicon generation and sequencing regarding detection of drug-resistant (AZT, 3TC, NVP) HIV-1 variants.

Pat No. *	Sample No.	Viral load (c/ml)	RTP (n)	RT region (aa pos)	ASPCR Resistance Mutation; %		Total reads	Mutant reads
1	1	1.13E+03	3	1–251	wt	-	-	-
2	2	3.26E+03	2	1–251	K70R	10	13228	1318
					E138A	97.6	13252	12937
	3	5.24E+04	5	1–237	Y181C	1.2	6379	78
3	4	1.42E+04	5	1–251	K70R	6.5	8267	536
					V108I	7.5	3964	299
	5	2.21E+04	5	1–251	K70R	2.0	3104	63
					V108I	2.0	1076	22
4	6	2.40E+04	5	1–251	K70R	9.6	7896	754
					E138K	3.6	3691	134
	7	4.63E+04	5	1–251	K70R	9.0	6960	626
					T215I	9.1	4580	416
5	8	3.47E+04	6	1–251	wt	-	-	-
	9	1.42E+03	2	145–251	wt	-	-	-
	10	5.44E+04	5	1–251	K65R	1.7	1665	28
	11	8.46E+04	3	1–251	wt	-	-	-
6	12	1.87E+04	3	1–251	wt	-	-	-
8	13	1.39E+05	6	1–251	K65R	1.1	1905	20
					K101E	6.3	1787	113
					K103N	7.1	1787	127
9	14	1.47E+03	0	n.a.	n.a.	-	-	-
	15	1.65E+04	4	1–251	K101E	3.6	1203	43
					K103R	1.2	1202	14
					V106M	3.7	1202	45
					G190A	1.5	3769	57
					P225H	1.1	3772	40
10	16	1.23E+04	3	1–251	K70R	5.4	1507	81
					V108I	3.9	1506	58
11	17	3.94E+03	6	1–251	K70R	1.0	1935	20
12	18	1.07E+03	3	1–237	V90I	4.4	1610	71
13	19	3.50E+04	4	1–237	wt	-	-	-
14	20	1.23E+03	3	1–170	wt	-	-	-
15	21	4.69E+03	3	1–170	V106I	2.0	1715	34
16	22	4.47E+03	5	1–251	K103N	41.9	845	354
					V106A	10.3	845	87
					Y181C	11.4	3151	358
					G190A	1.6	3150	49
	23	7.00E+03	2	1–251	K103N	3.6	3525	126
18	24	6.60E+03	0	n.a.	n.a.	-	-	-
19	25	9.60E+04	6	1–251	K65R	1.0	7478	74
					M184I	1.3	9141	117
					G190A	99.1	9141	9060
	26	6.50E+03	0	n.a.	n.a.	-	-	-
20	27	4.67E+05	6	1–251	K65R	1.0	9733	95
	28	2.76E+04	4	1–80,145–251	M184I	6.1	9556	582
21	29	7.92E+03	4	1–237	E138K	2.2	6006	131
22	30	4.87E+04	3	1–251	wt	-	-	-
23	31	5.74E+04	5	1–251	V108I	1.6	6939	111
24	32	1.63E+05	4	1–170	E138A	29.1	4840	1408
					E138G	3.9	4840	188
					E138R	3.9	4840	188
25	33	4.68E+05	5	1–251	wt	-	-	-
26	34	7.00E+03	2	145–251	Y188C	5.1	10500	534
27	35	2.48E+03	4	1–251	wt	-	-	-
28	36	1.17E+05	4	1–170	wt	-	-	-
29	37	5.32E+04	2	1–170	wt	-	-	-
30	38	1.75E+04	3	1–251	K65R	1.1	4331	46
31	39	4.63E+03	4	1–170	wt	-	-	-
32	40	9.13E+03	5	1–251	Y181C	2.0	6750	133
33	41	7.86E+04	5	1–251	K70R	1.0	6545	62
					K101E	6.9	3997	276

* according to Hauser *et al*. [10]

aa amino acid; c/ml copies per ml; coverage: min and max number of reads per position; UDS RTP (n) number of successfully generated overlapping amplicons out of six; n.a. not analyzed; wt wild type sequence

A total of 154/228 (68%) PCR amplicons was generated (median 4 RTPs/sample, IQR 3–5). Amplification of RTP4 (10/38) was significantly less successful compared to RTP1 (30/38), RTP2 (27/38), RTP3 (31/38), RTP5 (30/38), and RTP6 (26/38) (RTP4 vs RTP1-3/5-6: all *p<*0.001). Successful generation of amplicons significantly correlated with viral loads above 20,000 copies/ml (66/114 vs 88/114: *p* = 0.003). Amplification success did not depend on the HIV-1 subtype, because 107/156 RTPs were amplified from subtype C-isolates and 47/72 from subtype A1-isolates (*p* = 0.650) (Table 2).

The failure of RTP generation could be compensated in 20/33 samples by overlapping RTPs (data not shown). Analysis of the entire RT-genomic region (amino acids (aa) 1–251 of RT) was therefore possible for 25/38 samples. However, in 13 samples the RT-region was not fully covered: the 3'-RT-region downstream of the aa position 170 or 237 was missing in ten samples (no compensation for RTP4 and RTP6 or for RTP6 only, respectively) and the 5'-RT-region upstream of the aa position 145 was missing in three samples (no compensation of RTP1-4 or RTP 4–5, respectively).

A median number of 16,735 reads per sample was obtained (IQR 13,024–23,043), and drug resistance mutations were detected with coverages ranging from 845 to 13,252. In total, 45 drug-selected resistant variants were identified by amplicon-based UDS in 25 samples. Nine resistant variants with the AZT-selected mutations K70R and/or T215I were identified in eight samples at frequencies of 1.0% to 10.0%. Twenty-eight NVP-selected mutations (V90I, K101E, K103N/R, V106A/I/M, V108I, E138A/G/K/R, Y181C, Y188C, G190A or P225H) were detected in 19 samples at levels of 1.1% to 99.1%. Seven 3TC-selected mutations (K65R or M184I) were present in six samples at frequencies of 1.0% to 6.1% (Table 2). From a total of 45 resistance mutations, 18 (40%) were present at ASPCR key resistance positions, while 27 (60%) were detected at nine additional positions in RT not analysed by ASPCR. No drug-resistant variants were detected in 13 samples, but for nine of these samples presence of drug-resistant variants cannot be excluded due to missing regions in the RT (Table 2).

### Comparison of UDS with ASPCR and Sanger sequencing

For comparison of UDS and ASPCR, only key resistance mutations localized at RT positions K70, K103, Y181, M184, and T215 were considered. For comparison of UDS and Sanger sequencing, the positions V106 and K65 were also investigated.

Twenty-four HIV-1 resistance mutations were detected in 20 samples by ASPCR: 16x selected by AZT (11x K70R, 4x T215F, 1x T215Y), 6x selected by NVP (5x K103N, 1x Y191C), 2x selected by 3TC (2x M184V), while in 17 samples no key resistance mutations were detected (Tables 1 and 3).

**Table 3:** Comparison of UDS, ASPCR, and Sanger sequencing.

Sample No.	Viral load (c/ml)	Sanger Sequence	ASPCR Resistance Mutation, %		UDS Resistance Mutation, %		Total reads	Mutant reads
**Mutations detected by UDS and ASPCR (and/or Sanger)**								
2	3.26E+03	K70K/R	K70R	11	K70R	10.0	13228	1318
4	1.42E+04	K70K/R	K70R	14	K70R	6.5	8267	536
5	2.21E+04	wt	K70R	5.4	K70R	2.0	3104	63
6	2.40E+04	K70K/R	K70R	28	K70R	9.6	7896	754
7	4.63E+04	K70K/R	K70R	14	K70R	9.0	6960	626
8	3.47E+04	wt	K70R	2.0	K70R	0.3	7882	27
13	1.39E+05	wt	K103N	1.3	K103N	7.1	1787	127
16	1.23E+04	wt	K70R	4.9	K70R	5.4	1507	81
17	3.94E+03	wt	K70R	2.7	K70R	1.0	1935	20
22	4.47E+03	K103K/N	K103N	36	K103N	41.9	845	354
		Y181Y/C	Y181C	20	Y181C	11.4	3151	358
23	7.00E+03	K103K/N	K103N	12	K103N	3.6	3525	126
12	1.87E+04	wt	wt	-	wt	-	-	-
27	4.67E+05	wt	wt	-	wt	-	-	-
29	7.92E+03	wt	wt	**-**	wt	-	-	-
30	4.87E+04	wt	wt	-	wt	-	-	-
31	5.74E+04	wt	wt	-	wt	-	-	-
32	1.63E+05	wt	wt	-	wt	-	-	-
34	7.00E+03	wt	wt	-	wt	-	-	-
35	2.48E+03	wt	wt	-	wt	-	-	-
36	1.17E+05	wt	wt	-	wt	-	-	-
37	5.32E+04	wt	wt	-	wt	-	-	-
38	1.75E+04	wt	wt	-	wt	-	-	-
39	4.63E+03	wt	wt	-	wt	-	-	-
**Mutations detected by ASPCR (and/or Sanger) but not by UDS**								
1	1.13E+03	K70K/R	K70R	13	K70R	-	3363	<10
8	3.47E+04	wt	T215F	0.5	T215F	-	4142	0
9	1.42E+03	wt	T215F	0.5	T215F	-	11661	0
		K65K/R	n.a.	-	K65R	-	0	0
10	5.44E+04	wt	K70R	2.3	K70R	-	1665	0
11	8.46E+04	wt	T215F	0.7	T215F	-	11661	0
15	1.65E+04	wt	K103N	3.4	K103N	-	1202	0
18	1.07E+03	wt	T215F	0.8	T215F	-	1606	0
19	3.50E+04	wt	T215Y	3.9	T215Y	-	1487	0
20	3.40E+04	wt	K103N	2.1	K103N	-	144	0
21	4.69E+03	wt	K103N	3.4	K103N	-	1715	0
22	4.47E+03	wt	M184V	0.6	M184V	-	3151	0
23	7.00E+03	wt	K70R	4.0	K70R	-	5521	<10
28	2.76E+04	wt	M184V	0.6	M184V	-	9556	<10
**Mutations detected by UDS but not by ASPCR (and/or Sanger)**								
3	5.24E+04	wt	wt	-	Y181C	1.2	6379	78
7	4.63E+04	wt	n.a.	-	T215I	9.1	4580	416
15	1.65E+04	wt	n.a.	-	K103R	1.2	1202	14
22	4.47E+03	V106V/A	n.a.	-	V106A	10.3	845	87
25	9.60E+04	wt	n.a.	-	M184I	1.3	9141	117
28	2.76E+04	wt	n.a.	-	M184I	6.1	9556	582
33	4.68E+05	wt	wt	-	K70R	0.5	10756	52
40	9.13E+03	wt	wt	-	Y181C	2.0	6750	133
41	7.86E+04	wt	wt	-	K70R	1.0	6545	62

c/ml copies per ml; n.a. not analysed; wt wild type sequence (Sanger); wt no key resistance mutation detected

The presence of 12/24 (50%) ASPCR-detected resistance mutations could be reproduced by UDS. For eight of these, mutant variants were quantified at a lower proportion by UDS compared to ASPCR (median: 2.7 times lower; IQR 2.1–3.0). In one sample, the proportion of mutant variant was 5.5 times higher by UDS than by ASPCR (sample No. 13: 7.1% vs 1.3%). In three samples the proportions quantified by UDS and ASPCR were very similar, showing maximally 10% difference (0.1–1.1 times; sample No. 2: 10.0% vs 11%, sample No. 16: 5.4% vs 4.9%; sample No. 22: 41.9% vs 36%) (Table 3).

In 13/24 (54%) samples, the ASPCR-detected key resistance mutations could not be confirmed by UDS. All mutations but one were present at low (<5%; n = 5) or at very low (<1%; n = 6) frequencies. Notably, in two of these samples other resistance mutations were detected at the same position in the RT with a different aa substitution than that revealed by ASPCR (sample No. 15: K103R instead of K103N; sample No. 28: M184I instead of M184V) (Table 3).

For 13/17 samples, the absence of key resistance mutations as analysed by ASPCR was confirmed by UDS (inclusive sample No. 25 with M184I). However, in four cases very low proportions of key resistance mutations were detected by UDS: 0.5% K70R (sample No. 33) 1.0% K70R (sample No. 41), 1.2% Y181C (sample No. 3), and 2.0% (sample No. 40). The proportions of K70R quantified by UDS were close to the ASPCR detection limit (0.99% for K70R), whereas the proportions of Y181C quantified by UDS were above the ASPCR detection limit (0.35% for Y181C) [10] (Table 3).

All eight key resistance mutations detected by Sanger sequencing were confirmed by ASPCR, whereas UDS missed one mutation (sample No. 1: K70K/R). With two exceptions (both in sample No. 22), the proportions of drug-resistant variants were >11% by ASPCR and ≤11% by UDS. Of two additional mutations (sample No. 9: K65K/R, sample No. 22: V106V/A) detected by Sanger sequencing, V106A was identified by UDS at <11%, whereas the K65R could not be confirmed due to insufficient RTP coverage (Tables 2 and 3).

### Prediction of drug-resistance based on UDS and ASPCR results

Fourty-five drug resistance mutations were detected by UDS in 25 back-up samples of 20 women compared to 24 drug resistance mutations detected by ASPCR in 20 samples of 13 women (Table 4).

**Table 4:** Prediction of drug-resistance based on UDS and ASPCR results.

Pat No. *	Sample No.	UDS Resistance Mutation					ASPCR Resistance Mutation			Cum Res/ Pat (UDS)	Cum Res/ Pat (ASPCR)
1	1	-	-	-	-	-	K70R	-	-	s	AZT
2	2	K70R	E138A	-	-	-	K70R	-	-	AZT/NVP	AZT
	3	Y181C	-	-	-	-	-	-	-		
3	4	K70R	V108I	-	-	-	K70R	-	-	AZT/NVP	AZT
	5	K70R	V108I	-	-	-	K70R	-	-		
4	6	K70R	E138K	-	-	-	K70R	-	-	AZT/NVP	AZT
	7	K70R	T215I	-	-	-	K70R	-	-		
5	8	-	-	-	-	-	K70R	T215F	-	3TC	AZT
	9	-	-	-	-	-	T215F	-	-		
	10	K65R	-	-	-	-	K70R	-	-		
	11	-	-	-	-	-	T215F	-	-		
6	12	-	-	-	-	-	-	-	-	s	s
8	13	K65R	K103N	K101E	-	-	K103N	-	-	3TC/NVP	NVP
9	15	K101E	K103R	V106M	G190A	P225H	K103N	-	-	NVP	NVP
10	16	K70R	V108I	-	-	-	K70R	-	-	AZT/NVP	AZT
11	17	K70R	-	-	-	-	K70R	-	-	AZT	AZT
12	18	V90I	-	-	-	-	T215F	-	-	NVP	AZT
13	19	-	-	-	-	-	T215Y	-	-	s	AZT
15	21	V106I	-	-	-	-	K103N	-	-	NVP	NVP
16	22	K103N	V106A	Y181C	G190A	-	M184V	K103N	Y181C	NVP	AZT/NVP/3TC
	23	K103N	-	-	-	-	K103N	K70R	-		
19	25	M184I	K65R	G190A	-	-	-	-	-	3TC/NVP	s
20	27	K65R	-	-	-	-	-	-	-	3TC	3TC
	28	M184I	-	-	-	-	M184V	-	-		
21	29	E138K	-	-	-	-	-	-	-	NVP	s
22	30	-	-	-	-	-	-	-	-	s	s
23	31	V108I	-	-	-	-	-	-	-	NVP	s
24	32	E138A/G/R	-	-	-	-	-	-	-	NVP	s
25	33	-	-	-	-	-	-	-	-	s	s
26	34	Y188C	-	-	-	-	-	-	-	NVP	s
27	35	-	-	-	-	-	-	-	-	s	s
28	36	-	-	-	-	-	-	-	-	s	s
29	37	-	-	-	-	-	-	-	-	s	s
30	38	K65R	-	-	-	-	-	-	-	3TC	s
31	39	-	-	-	-	-	-	-	-	s	s
32	40	Y181C	-	-	-	-	-	-	-	NVP	s
33	41	K70R	K101E	-	-	-	-	-	-	AZT/NVP	s

* according to Hauser *et al*. [10]

s susceptible; Cum Res/Pat Cumulative resistance per patient

Based on the UDS results, 69% (20/29) of women harboured drug-resistant HIV variants (cumulative resistance of all follow up samples, Table 4): AZT-resistance mutations were identified in six, NVP-resistance in 16 and 3TC-resistance in five women, resulting in mono-resistance to AZT in 3% (1/29), NVP in 31% (9/29), and 3TC in 10% (3/29) of the women. Dual-resistant virus populations were present in 24% (7/29), either selected by AZT and NVP (n = 5) or by NVP and 3TC (n = 2). No multi-resistance (AZT/NVP/3TC) was detected by UDS. Wild-type virus was present in 9/29 women (31%) (Table 4).

Based on the ASPCR results, drug-resistance was found to affect 45% (13/29) of women. AZT-resistance mutations were detected in ten, NVP-resistance in four, and 3TC-resistance in two women. These mutations resulted in mono-resistance to AZT in 31% (9/29), NVP in 10% (3/29), and 3TC in 3% (1/29) of women. Multi-resistant virus (AZT/NVP/3TC) was detected in one woman. No key resistance mutation was detected by ASPCR in 52% (15/29) of women (Table 4).

Resistant HIV-1 variants in proportions below 5% of the total viral population were present in 45% (9/29) and in 57% (8/14) of women according to the quantification by UDS and ASPCR, respectively (Tables 1 and 2).

## Discussion

Forty-one samples of 31 Tanzanian women pre-screened in a previous study [10] by ASPCR for HIV-1 genomes carrying key resistance mutations selected by AZT, 3TC, and NVP were investigated by UDS to compare the presence of drug-resistance mutations and to identify additional antiretroviral regimen-selected resistance mutations in the HIV-1 RT-region. The amplicon-based UDS and the ASPCR were performed using independently isolated RNA of two different aliquots of the same plasma sample. The results obtained using the two methods were compared.

In the amplicon-based UDS, 45 AZT-, 3TC-, and/or NVP-selected resistance mutations were detected in 25 of 37 back-up samples collected from 29 mothers at and after delivery. Only half of the ASPCR-detected resistance mutations identified previously [10] could be reproduced by UDS using back-up samples. The potential reasons for this discrepancy are discussed below.

The generation of amplicons is an essential and critical step in the UDS-workflow. Applying the HIV-1 primers designed for 454 FLX sequencing by Roche, only two-thirds of the potential amplicons were amplified successfully. The significantly lower RTP4 primer-efficiency in PCR is probably the consequence of the larger fragment size as compared to the other amplicons. Statistical analysis revealed a highly significant correlation between viral load and amplicon generation of RTP1-6, which was also shown for other UDS assays [25, 26]. Samples with viral loads above 20,000 copies/ml were amplified more efficiently than samples with viral loads below or equal to 20,000 copies/ml. In fact, all three samples failing RTP amplification had low viral loads (<7,000 copies/ml). Likewise, five samples with incomplete RT coverage (RT codon 1–170, 1–236, or 145–251 instead of codon 1–251) had very low viral loads (<5,000 copies/ml), and therefore no sequence was obtained in the respective 3'-region or 5'-region of RT. The primer design can be considered as generic for HIV-1 subtypes A and C, because both subtypes were amplified with the same success rate. The impact of the larger RTP4 fragment size as well as the correlation between viral load and amplicon generation has also been discussed by St. John *et al*. [11] in line with the follow-up international multicentre collaborative study. According to our data, a minimum input of 20,000 copies/ml and an extraction volume of 500 μl plasma should be considered for UDS to guarantee successful amplification of all six amplicons, confirming the 454 recommendation of using the equivalent of at least 2,000 copies per 10 μl RNA [11, 12].

The sensitivity of UDS depends on the mutation-specific error rate, the read coverage at the drug resistance position, and the input viral load of the sample [14, 15, 17–19, 33]. A coverage of at least 50 mutant reads in a total of 5000 reads at the position of interest is recommended to reliably detect minorities at 1% [34]. However, in our hands the UDS coverage was below 5000 reads for 29/45 drug resistance mutations. Because the site-specific error rate at all detected drug resistance mutations was below 0.25% in control samples, we permitted a frequency of 1% (represented by a minimum number of 10 mutant reads in a total of 1000 reads) to identify unknown drug-resistant minor variants [16, 25]. Nevertheless, it should be stressed that these settings were used for research only in this study, and that this procedure might be inappropriate for resistance testing and decisions concerning therapy.

Only 12/24 drug resistance mutations detected by ASPCR were confirmed by UDS at predominantly lower frequencies. Indeed, proportions quantified by UDS were on average three times lower than those quantified by ASPCR. Similarly, all resistance mutations identified by Sanger sequencing [10] were quantified by UDS with proportions of ≤11% but >11% by ASPCR, although the detection limit of Sanger sequencing is well known to be approximately 20% [5, 6]. Therefore, UDS seems to underestimate the true proportion of mutant variants, which might be a reason for the failure of UDS in reproducing minor variants with frequencies below 5% or even 1% as determined by ASPCR. Another study from Delobel *et al*. [35], who compared the performance of 454 UDS and ASPCR to detect the K103N resistance mutation, also found ASPCR to be more sensitive. The overall concordance of UDS and ASPCR was therefore 61.0% (25/41) for the absence (n = 13/17) and presence (n = 12/24) of resistance mutations.

Discrepant results between the samples used in the previous ASPCR analysis and the back-up samples used in the current UDS analysis can be explained by the fact that the proportion of the resistant variant was near the detection limit. At concentrations close to the detection limit, the amplification of independent aliquots drawn from the same sample will lead to inconsistent results according to the Poisson distribution [14]. Furthermore, more than a half (7/13) of the samples containing minor drug-resistant HIV-1 variants according to ASPCR not detected by UDS had viral loads below 20.000 copies/ml and for 5/7 samples only 2–3 RTP amplicons could be generated. In the ASPCR a shorter PCR fragment (644 bp) was amplified, resulting in a more sensitive detection limit of 650 copies/ml [10] as compared to UDS. In addition, in two strains with key resistance mutations detected by ASPCR, a mutation with a different amino acid substitution at the same position was identified by UDS. This presumes that a mutant-specific ASPCR primer, which was designed to distinguish between a specific mutation and the wild type, could misprime at the same position and give positive signals for any “not wild-type” nucleotide. This may result in a qualitatively correct result (mutant) but the type and quantity of mutant would not be valid. Indeed, ASPCR may be prone to errors as a result of polymorphisms in the primer binding sites that limit the success of the assay [10, 27]. Considering this non-wild-type amino acid substitutions as concordant between UDS and ASPCR, the overall concordance of UDS and ASPCR would increase to 65.9% (25/41) for the absence (n = 13/17) and presence (n = 12/24) of resistance mutations.

In the present study, the total number of resistance mutations detected by UDS was almost twice that of the number detected by ASPCR (45 vs 24). However, 60% of these mutations were present at nine additional positions in the RT which were not covered by the ASPCR assays applied here. The presence of NVP- and 3TC-selected resistance mutations was therefore underestimated by ASPCR as compared to UDS (NVP: 4 vs 15, 3TC: 2 vs 5). Consequently, UDS revealed dual-resistant HIV-populations in 45% of women compared to 6% of women with dual/multi-resistant HIV-populations identified by ASPCR. Furthermore, the more sensitive detection limit of the ASPCR (<1%) compared to UDS (≥1%) resulted in higher proportions of minor variants <5% detected by ASPCR than by UDS (57% vs 45%). Resistance mutations present in low frequencies such as the AZT resistance mutations, in particular the minor mutation T215Y/F that occurred at low proportions of <1% due to the higher genetic barrier of two amino acid substitutions, were therefore underestimated by UDS as compared to ASPCR (6 vs 10). The clinical relevance of drug-resistant minor variants is still a matter of debate and their impact on treatment outcome may depend on the type of drug resistance mutation and the viral load [8, 9, 33–35]. Nevertheless, a recently published Europe-wide case-control study using 454 UDS demonstrated that drug-resistant minor variants more than double the risk of virological failure for first-line therapy including NNRTI [36], which highlights the need for more sensitive drug-resistance testing methods also in clinical routine.

## Conclusions

Despite the small number of patient samples analysed in this comparative study, some conclusions can be drawn about the general benefits or limitations of the two methods.

In the present work, by UDS almost twice the number of mutations as compared to the ASPCR were detected at nine additional positions. This fact reveals the limitation of ASPCR in its restriction to only one mutation that can be targeted per reaction. Analysing an amplicon by UDS provides detailed information of all resistance mutations present in the complete genome fragment sequenced and thus provides more comprehensive predictions of drug-resistance for subsequent antiretroviral treatments.

In contrast, the UDS outcome presented here was strongly limited by the less sensitive PCR and the lower detection limit for resistance mutations. A minimum input of 20,000 copies/ml is a prerequisite for successful amplification and for sufficient sequence coverage and, in turn, a reliable cut-off to detect mutant variants at 1% proportions.

Both methods render the detection of drug-resistant virus populations more sensitive than Sanger sequencing and can allow their detection at frequencies as low as 1%. Despite the fact that the clinical relevance of these minor variants for treatment response is not yet established, sensitive detection methods are essential for assessing the prevalence of drug-resistant minor variants and their impact on treatment outcome. Methodological advances in UDS-technology will definitively offer the ability to routinely monitor the presence of minor variants in the HIV quasispecies.
